# CeO_2_ Nanoparticles Seed Priming Increases Salicylic Acid Level and ROS Scavenging Ability to Improve Rapeseed Salt Tolerance

**DOI:** 10.1002/gch2.202200025

**Published:** 2022-05-19

**Authors:** Mohammad Nauman Khan, Yanhui Li, Chengcheng Fu, Jin Hu, Linlin Chen, Jiasen Yan, Zaid Khan, Honghong Wu, Zhaohu Li

**Affiliations:** ^1^ MOA Key Laboratory of Crop Ecophysiology and Farming System in the Middle Reaches of the Yangtze River College of Plant Science and Technology Huazhong Agricultural University Wuhan 430070 China; ^2^ Hongshan Laboratory Wuhan Hubei 430070 China; ^3^ College of Agronomy and Biotechnology China Agricultural University Beijing 100083 China

**Keywords:** gene expression, nanoceria seed priming, ROS scavenging, salicylic acid, salinity

## Abstract

Soil salinity is a major issue limiting efficient crop production. Seed priming with nanomaterials (nanopriming) is a cost‐effective technology to improve seed germination under salinity; however, the underlying mechanisms still need to be explored. Here, polyacrylic acid coated nanoceria (cerium oxide nanoparticles) (PNC, 9.2 nm, −38.7 mV) are synthesized and characterized. The results show that under salinity, PNC priming significantly increases rapeseed shoot length (41.5%), root length (93%), and seedling dry weight (78%) compared to the no‐nanoparticle (NNP) priming group. Confocal imaging results show that compared with NNP group, PNC priming significantly reduces reactive oxygen species (ROS) level in leaf (94.3% of H_2_O_2_, 56.4% of ^•^O_2_^−^) and root (38.4% of H_2_O_2_, 41.3% of ^•^O_2_^−^) of salt stressed rapeseed seedlings. Further, the results show that compared with the NNP group, PNC priming not only increases salicylic acid (SA) content in shoot (51.3%) and root (78.4%), but also upregulates the expression of SA biosynthesis related genes in salt stressed rapeseed. Overall, PNC nanopriming improved rapeseed salt tolerance is associated with both the increase of ROS scavenging ability and the increase of salicylic acid. The results add more information to understand the complexity of mechanisms behind nanoceria priming improved plant salt tolerance.

## Graphical abstract

Seed nanopriming to improve plant stress tolerance is an approach with good potential in agricultural application. Herein, it is shown that besides the commonly employed mechanisms i.e., reactive oxygen species (ROS) scavenging, seed priming with CeO_2_ nanoparticles can also modulate phytohormone levels, such as absicic acid, gibberellic acid 3, and especially the salicylic acid content, to induce salt tolerance in rapeseed.

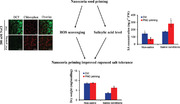

## 1 Introduction

Salinity affects more than 20 million hectares of the total world's arable land, resulting in penalty of crop yield and quality.^[^ ^1^ ^]^ Due to factors such as poor irrigation management, human activities, and climate change, the trend of soil salinization is increasing.^[^ ^2^, ^3^ ^]^ Plants are known to respond to salt stress in two phases: 1) high salinity induced osmotic stress, and 2) the over‐accumulation of ions such as sodium and chloride caused ionic toxicity.^[^ ^4^, ^5^ ^]^ Besides osmotic stress and ion toxicity of salinity on plants, the accumulation of reactive oxygen species (ROS) such as superoxide (^•^O_2_^−^), hydrogen peroxide (H_2_O_2_), hydroxyl radical (OH^•^), and singlet oxygen (^1^O_2_) could lead to oxidative stress. The oxidative stress causes adverse effects such as the oxidation of lipids and proteins, reduction of enzymatic activities, and DNA damage.^[^ ^5^, ^6^ ^]^

Rapeseed (*Brassica napus* L.) is a known important oilseed crop.^[^ ^7^ ^]^ However, environmental stresses such as drought, salinity, and waterlogging significantly reduce the biomass, seed yield, and oil production of rapeseed.^[^ ^8^, ^9^, ^10^ ^]^ For example, salinity delays seed germination and limits seed germination rates.^[^ ^11^, ^12^ ^]^ Salt stress significantly reduces the germination rate of rapeseed, primarily by disruption of seed water uptake (imbibition),^[^ ^5^ ^]^ reduction of the seed reserves utilization,^[^ ^13^ ^]^ inactivation of enzymes activities,^[^ ^14^ ^]^ and disturbance of hormonal balance.^[^ ^15^ ^]^ To achieve efficient seed germination, seed priming is regarded as a convenient, low‐cost, and effective approach.^[^ ^16^, ^17^ ^]^ Enhancement of seed germination and seedling growth with nanoparticles have recently become a new potential strategy in seed priming technology.^[^ ^18^, ^19^ ^]^ Seed nanopriming is a technique in which seeds are primed with nanoparticles. It serves to be a useful approach to enhancing seedling establishment, crop yields, seed quality and crop stress tolerance,^[^ ^20^ ^]^.^[^ ^21^ ^]^ For example, Fe_2_O_3_ nanoparticles were used for priming of wheat,^[^ ^22^ ^]^ sorghum,^[^ ^23^ ^]^ and watermelon^[^ ^24^ ^]^ seeds. ZnO nanoparticles were used for seed priming of wheat^[^ ^25^ ^]^ and peanut.^[^ ^26^ ^]^ Seed priming with silver nanoparticles were tested in rice^[^ ^21^ ^]^ and broad bean.^[^ ^27^ ^]^ Nonetheless, previous studies provided limited information regarding the role of phytohormones in seed nanopriming, with only reports on abscisic acid (ABA), gibberellins (GA), indole‐3‐butyric acid, 1‐naphthalene acetic acid, and 6‐benzylaminopurine contents.^[^ ^28^, ^29^, ^30^ ^]^ Other hormones such as BR (brassinosteroids), JA (jasmonic acid), and SA (salicylic acid) also play a role in seed germination.^[^ ^31^, ^32^, ^33^ ^]^ Investigating the role of common phytohormones besides ABA and GA in nanoprimed seeds is of interest to better understand the complexity of mechanisms underlying seed nanopriming.

Cerium oxide nanoparticles (nanoceria, CeO_2_‐NPs) are a potent scavenger of reactive oxygen species (ROS) due to catalytic scavenging properties and thus are widely used in industry, medical research, and plant science.^[^ ^34^, ^35^, ^36^ ^]^ Previous study reported that CeO_2_‐NPs coated with polyvinylpyrrolidone (PVP) (55.6 nm, −51.8 mV) improved rapeseed salt tolerance by increasing photosynthetic apparatus efficiency and thus resulted in higher biomass.^[^ ^37^ ^]^ Another mechanism regarding CeO_2_‐NPs improved rapeseed salt tolerance is that the CeO_2_‐NPs altered Na^+^ fluxes via shortening root apoplastic barriers, transported more Na^+^ to shoots, and reduced Na^+^ accumulation in roots.^[^ ^38^ ^]^ Similarly, the results of our previous study revealed that CeO_2_‐NPs coated with polyacrylic acid (PNC) (10 nm, −17 mV, scavenged hydroxyl radical, sustained higher potassium (K^+^) content in mesophyll and thus improved *Arabidopsis* salt tolerance.^[^ ^39^ ^]^ However, these studies mainly focused on the post‐germination mechanisms involved in CeO_2_‐NPs induced stress tolerance in different crop species. The mechanisms, especially the role of phytohormones, underlying nanoceria seed priming improved plant salt tolerance are still rare. Soaking seeds with nanoceria (2.1 nm, −51.7 mV) improved cotton salt tolerance via reducing ROS accumulation in seedling roots and modulated ion homeostasis.^[^ ^40^ ^]^ Our previous study revealed that PNC priming in *Brassica napus* significantly affected seed water uptake, upregulated *AMY1* and *AMY2* genes to improve α‐amylase activities, and ultimately the content of total soluble sugar in seeds during the imbibition period.

In this study, the role of nanoceria priming in improving rapeseed salt tolerance and its underlying mechanisms were investigated. ROS levels in salt‐stressed rapeseeds primed with nanoceria or the buffer control were compared. Then, under salinity, the possible changes of phytohormones, i.e., abscisic acid (ABA), gibberellic acid (GA_3_), jasmonic acid (JA), indole acetic acid (IAA), and salicylic acid (SA), in nanoceria primed rapeseed were investigated. Based on the obtained results, among the measured phytohormones, SA was chosen for further study. SA inhibitor was used to further validate the role of SA in PNC improved rapeseed salt tolerance. The expression level of genes related to SA biosynthesis was monitored. To the best of our knowledge, this could be the first study reporting the role of SA in nanoceria‐priming improved rapeseed salt tolerance.

## 2 Results

### 2.1 Characterization of PNC

**Figure 1:**
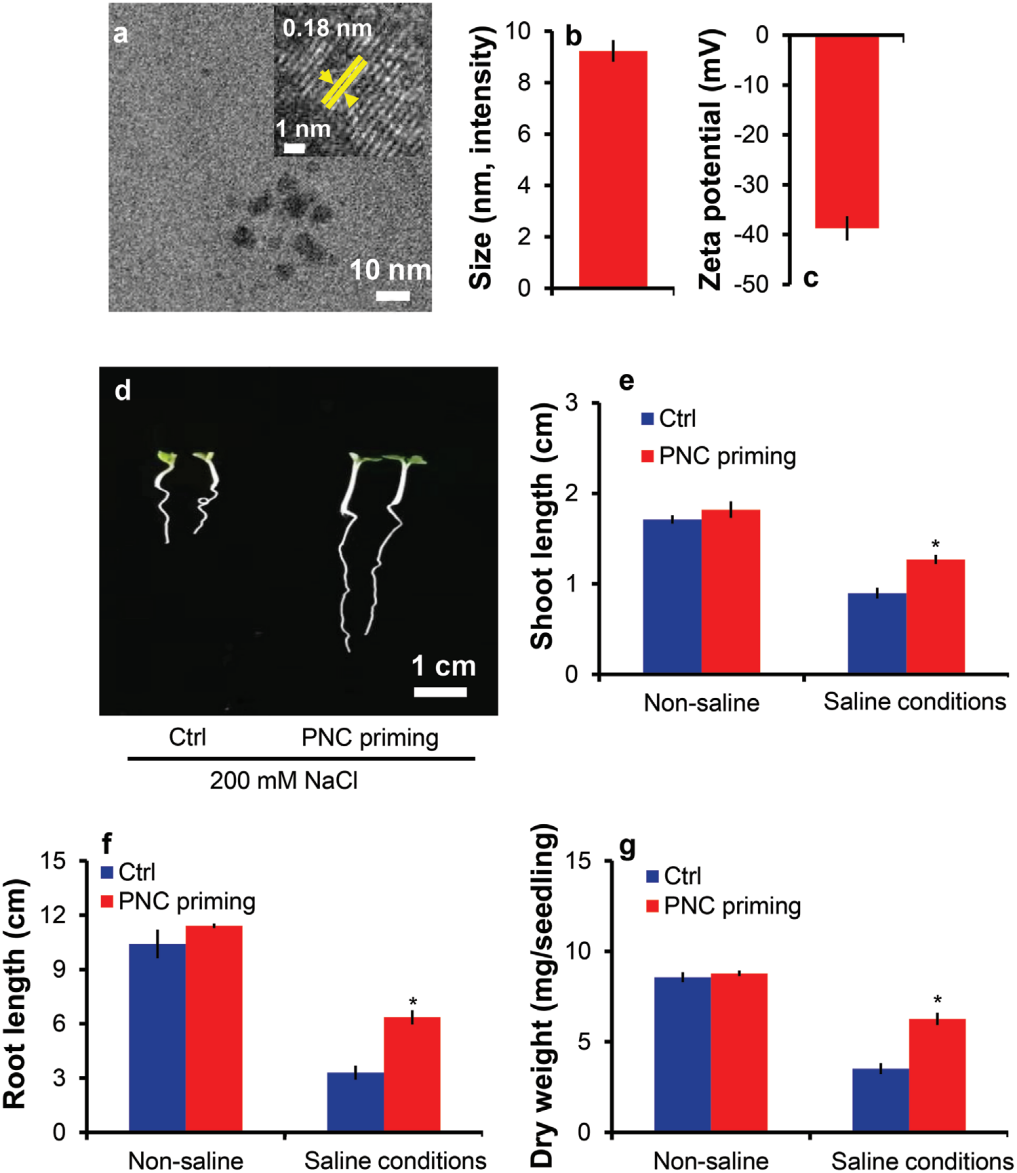
PNC characterization and the effect of PNC nanopriming on seedling growth of rapeseed under salinity. a) TEM image of PNC, b) PNC size by intensity, c) PNC zeta potential, d) phenotype of rapeseed under 200 × 10^−3^ m NaCl stress, e,f) effect of PNC priming on shoot and root length under non‐saline and saline growth conditions, and g) effect of PNC priming on seedling dry weight under non‐saline and saline growth conditions. The significance between the different treatments at *P* < 0.05 is indicated with * on the vertical bars. One batch was taken as one biological replicate (*n* = 3), while the error bars show the standard error of three biological replicates.

The absorbance curve of PNC showed a clear peak at 271 nm (Figure S1, Supporting Information). According to TEM (transmission electron microscopy) images, the average PNC core size was 4.1 ± 0.3 nm (**Figure** **1**a). The average size of PNC by intensity was 9.2 ± 0.4 nm (Figure 1b), and the average zeta potential was −38.7 ± 2.4 mV, according to data from a dynamic light scattering device (Malvern Zetasizer, Nano) (Figure 1c).

### 2.2 PNC Priming Improved Rapeseed Phenotype

Under normal growth conditions, PNC priming had no significant effect on rapeseed shoot and root lengths compared to no‐nanoparticle priming (NNP group) (Figure 1e,f). However, 200 × 10^−3^ m salt stress significantly reduced the shoot and root lengths (Figure 1d). Nonetheless, PNC priming increased shoot (41.5% increase, 1.26 ± 0.02 vs 0.89 ± 0.05 cm) and root (93% increase, 6.36 ± 0.22 vs 3.29 ± 0.07 cm) lengths under salt stress as compared to the NNP priming (Figure 1e,f) (*P* < 0.05). Due to the significant increase in shoot and root lengths, PNC priming markedly increased dry weight (6.26 ± 0.33 vs 3.52 ± 0.14 mg per seedling, 78% increase) compared to NNP priming (Figure 1g).

### 2.3 PNC Nanopriming Alleviates ROS Level in Leaf and Root Cells of Rapeseeds

**Figure 2:**
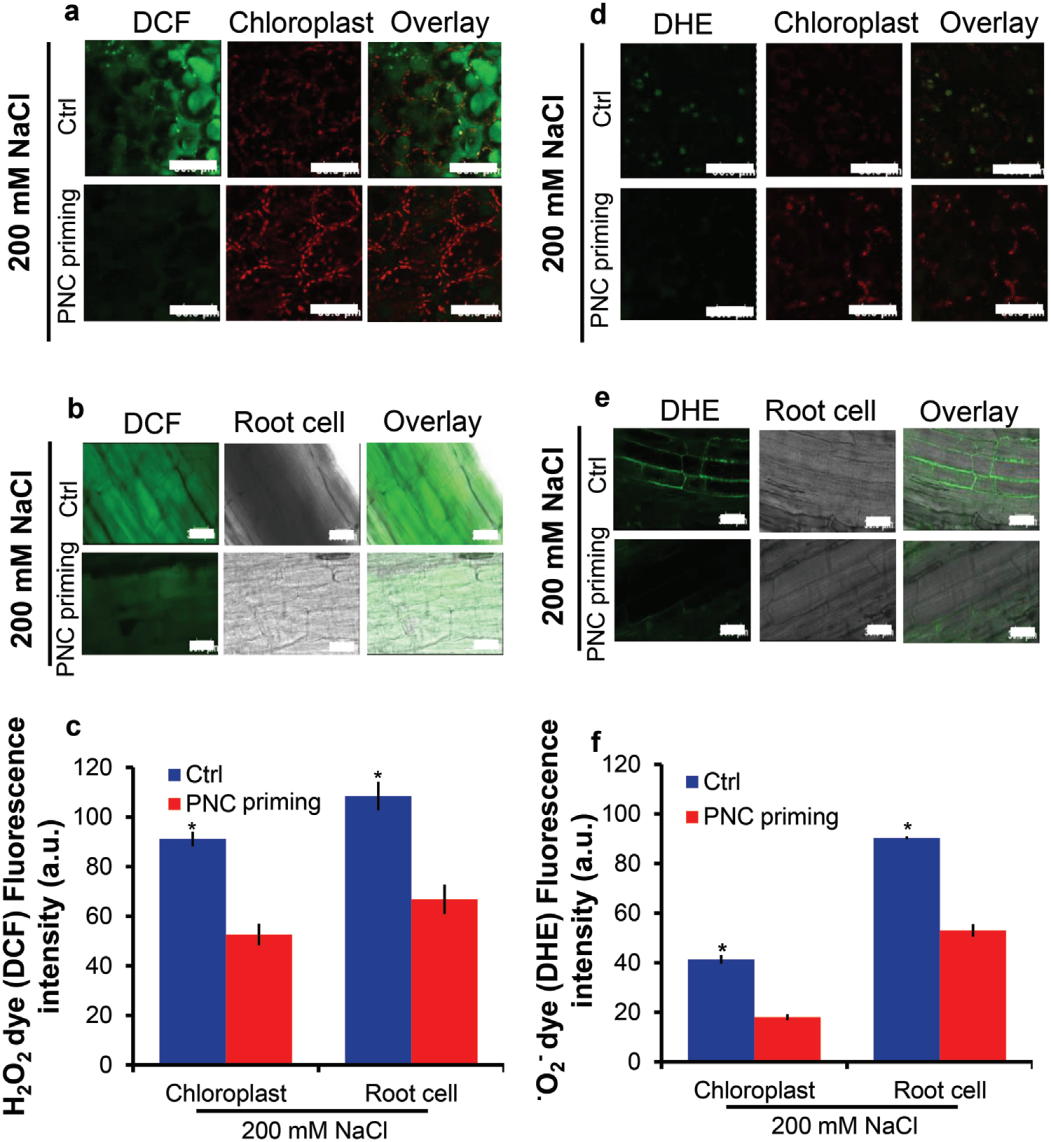
ROS level in leaf and root cells of PNC primed rapeseed under salinity. a,b) confocal imaging of hydrogen peroxide (H_2_O_2_) scavenging in leaf cells of rapeseed seedling leaves and root cells by PNC under saline growth conditions, c) the fluorescence intensity of H_2_O_2_ dye (DCF) in rapeseed seedling leaves and root cells under saline growth conditions, d,e), confocal imaging of superoxide anion (^•^O_2_^−^) scavenging in leaf cells of rapeseed seedling leaves and root cells by PNC under saline growth conditions, and f) the fluorescence intensity of ^•^O_2_^−^ dye (DHE) rapeseed seedling leaves and root cells under saline growth conditions. The significance between the different treatments at *P* < 0.05 is indicated with * on the vertical bars. Scale bar: 50 µm.

**Figure 3:**
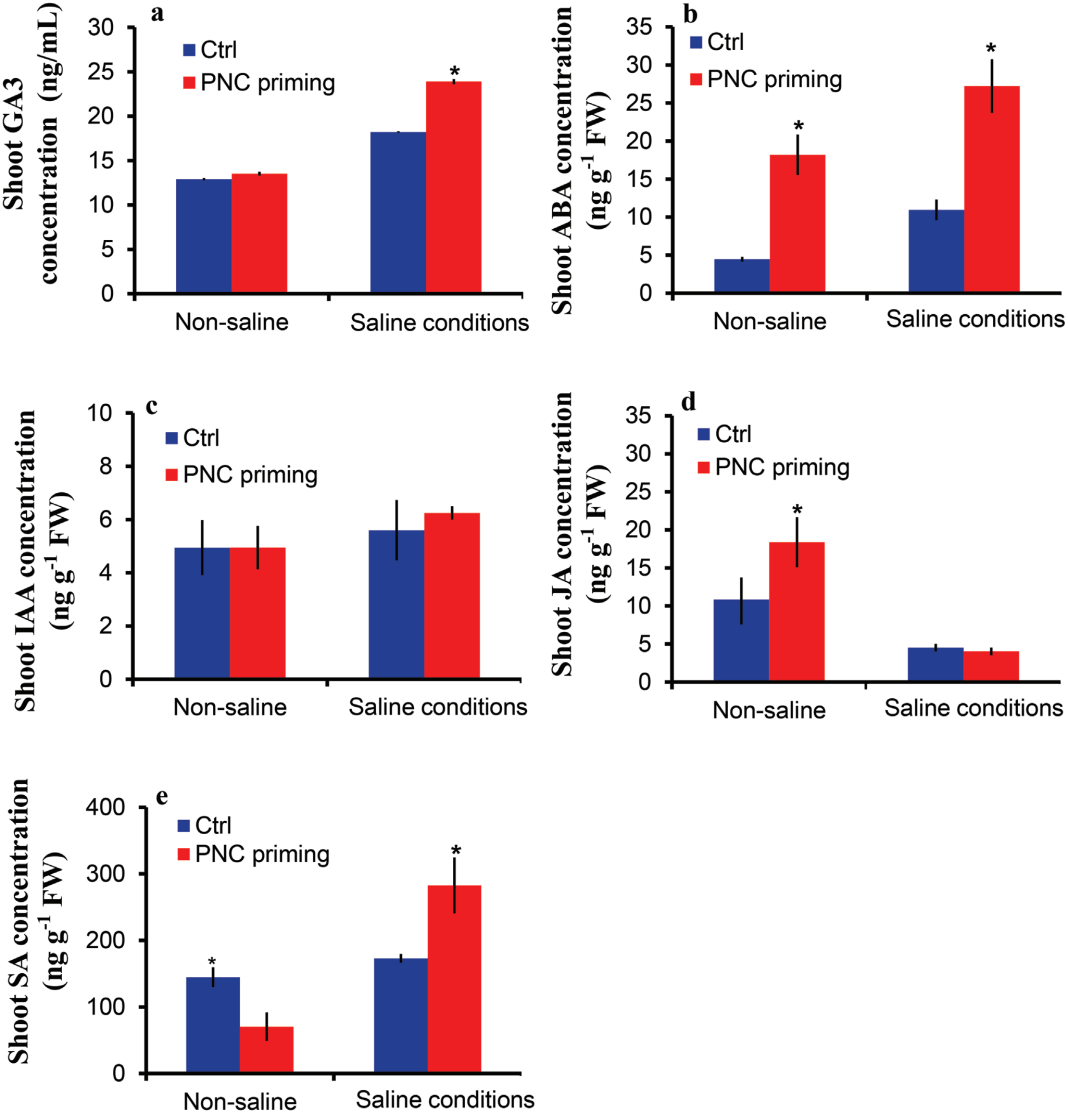
Hormonal changes in shoot of rapeseed primed with PNC. a) GA_3_ concentration shoot of rapeseed seedlings established from seeds primed with or without PNC under non‐saline (7 days) conditions, b) ABA concentration shoot of rapeseed seedlings established from seeds primed with or without PNC under non‐saline (7 days) conditions, c) IAA concentration in shoot of rapeseed seedlings established from seeds primed with or without PNC under non‐saline (7 days) conditions, d) JA concentration shoot of rapeseed seedlings established from seeds primed with or without PNC under non‐saline (7 days) conditions, and e) SA concentration in shoot of rapeseed seedlings established from seeds primed with or without PNC under non‐saline (7 days) conditions. The significance between the different treatments at *P* < 0.05 is indicated with * on the vertical bars. One batch was taken as one biological replicate (*n* = 3), while the error bars show the standard error of three biological replicates.

The amount of H_2_O_2_ in rapeseed cotyledon leaf and root cells was confirmed by staining with H_2_DCFDA (**Figure** **2**a,b), a ROS indicator that converts to its fluorescent DCF form upon reaction with ROS. Similarly, the amount of superoxide anion (^•^O_2_^−^) was detected with DHE (fluorescent product 2‐hydroxyethidium) (Figure 2d,e). The fluorescence of DHE increases upon reaction with superoxide anion (^•^O_2_^−^). Under 200 × 10^−3^ m salt stress conditions, PNC priming reduced the intensity of H_2_O_2_ (indicated by DCF fluorescent dye) accumulation in the cotyledon leaf by 94.3% (52.6 ± 5.7 vs 91.1 ± 2.9) and 38.4% (68.8 ± 5.9 vs 108.4 ± 4.3) in the root cells (Figure 2c). As compared to the control group, PNC priming reduced the accumulation of ^•^O_2_^−^ (indicated by DHE fluorescent dye) by 56.4% in the cotyledon leaf (18 ± 0.5 vs 41.3 ± 1.7) and 41.3% (53 ± 2.5 vs 90.3 ± 1.2) in the root cells, respectively, under 200 × 10^−3^ m NaCl stress (**Figure** **3**f). However, there was no significant difference between PNC priming and the control group for DCF and DHE intensities under normal growth conditions (Figures S2 and S3, Supporting Information).

### 2.4 PNC Nanopriming Modulates Hormonal Homeostasis in the Rapeseed Seedlings Shoot and Root to Combat Salt Stress

There was no significant difference in shoot GA_3_ concentration between NNP priming, PNC priming, and PNC priming under non‐saline conditions (Figure 3a). However, PNC priming had the highest shoot GA_3_ concentration (23.9 ± 0.2 vs 18.2 ± 0.07 ng g^‐−1^ FW, 31% increase) compared to the control group under saline conditions. Shoot ABA concentration was increased by 307% (18.18 ± 2.6 vs 4.46 ± 0.31 ng g^−1^ FW) and 149% (27.2 ± 3.5 10.9 ± 1.3 ng g^−1^ FW) under non‐saline and saline conditions, respectively, by PNC priming as compared to NNP priming (Figure 3b). Under non‐saline and saline conditions, no significant difference in shoot IAA concentration was recorded between NNP and PNC priming (Figure 3c). PNC priming increased shoot JA concentration by 69.3% (18.36 ± 3.2 vs 10.84 ± 2.8 ng g^−1^ FW) under non‐saline conditions with no significant effect on shoot JA concentration under saline conditions as compared to the NNP group (Figure 3d). Interestingly, PNC priming decreased shoot SA concentration by 51.3% (70.43 ± 21.29 vs 144.74 ± 15. 17 ng g^−1^ FW) under non‐saline conditions compared to the control treatment (Figure 3e). However, under saline conditions, an increase of 63.2% (282.5 ± 42.3 vs 173.03 ± 6.57 ng g^−1^ FW) in shoot SA concentration was observed for PNC priming as compared to NNP priming.

**Figure 4:**
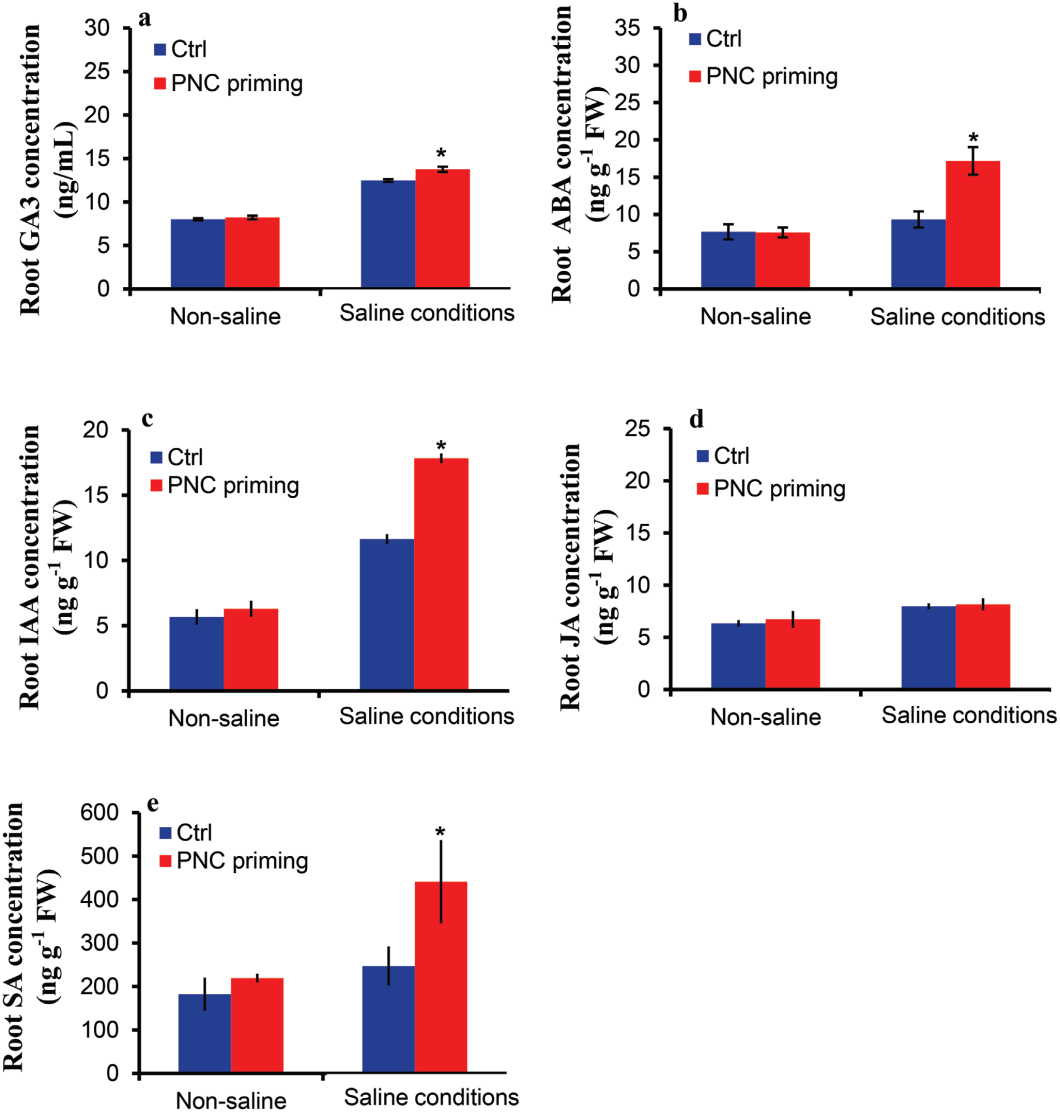
Hormonal changes in root of rapeseed primed with PNC. a) GA_3_ concentration in root of rapeseed seedlings established from seeds primed with or without PNC under 200 × 10^−3^ m NaCl stress (7 days) conditions, b) ABA concentration in root of rapeseed seedlings established from seeds primed with or without PNC under 200 × 10^−3^ m NaCl stress (7 days) conditions, c) IAA concentration in root of rapeseed seedlings established from seeds primed with or without PNC under 200 × 10^−3^ m NaCl stress (7 days) conditions, d) JA concentration in root of rapeseed seedlings established from seeds primed with or without PNC under 200 × 10^−3^ m NaCl stress (7 days) conditions, and e) SA concentration in root of rapeseed seedlings established from seeds primed with or without PNC under 200 × 10^−3^ m NaCl stress (7 days) conditions. The significance between the different treatments at *P* < 0.05 is indicated with * on the vertical bars. One batch was taken as one biological replicate (*n* = 3), while the error bars show the standard error of three biological replicates.

Under non‐saline conditions, PNC priming had no significant effect on the root GA_3_ concentration, while PNC priming increased root GA_3_ concentration by 10.28% (13.75 ± 0.3 vs 12.4 ± 0.15 ng g^−1^ FW) (**Figure** **4**a). Similarly, root ABA concentration was increased up to 84.3% (17.18 ± 1.8 vs 9.32 ± 1.07 ng g^−1^ FW) by PNC priming under saline conditions with no significant difference under non‐saline conditions as compared to NNP priming (Figure 4b). Compared with NNP priming, PNC priming had a significant effect on the root IAA concentration (17.8 ± 0.3 vs 11.6 ± 0.6 ng g^−1^ FW, 53% increase) under saline conditions, with no significant effect under non‐saline conditions (Figure 4c). There was no significant difference in root JA concentration between PNC priming and NNP priming under non‐saline and saline conditions (Figure 4d). Nonetheless, the priming treatments did not significantly affect the root SA concentration under non‐saline conditions (Figure 4e). However, under saline conditions, PNC priming significantly increased the root SA concentration by 78.4% (440.9 ± 55.3 vs 247.12 ± 25.9 ng g^−1^ FW) as compared to NNP priming.

### 2.5 Exogenously Supplied Salicylic Acid (SA) Improved Rapeseed Germination and Biomass under Salinity Stress via Scavenging ROS

**Figure 5:**
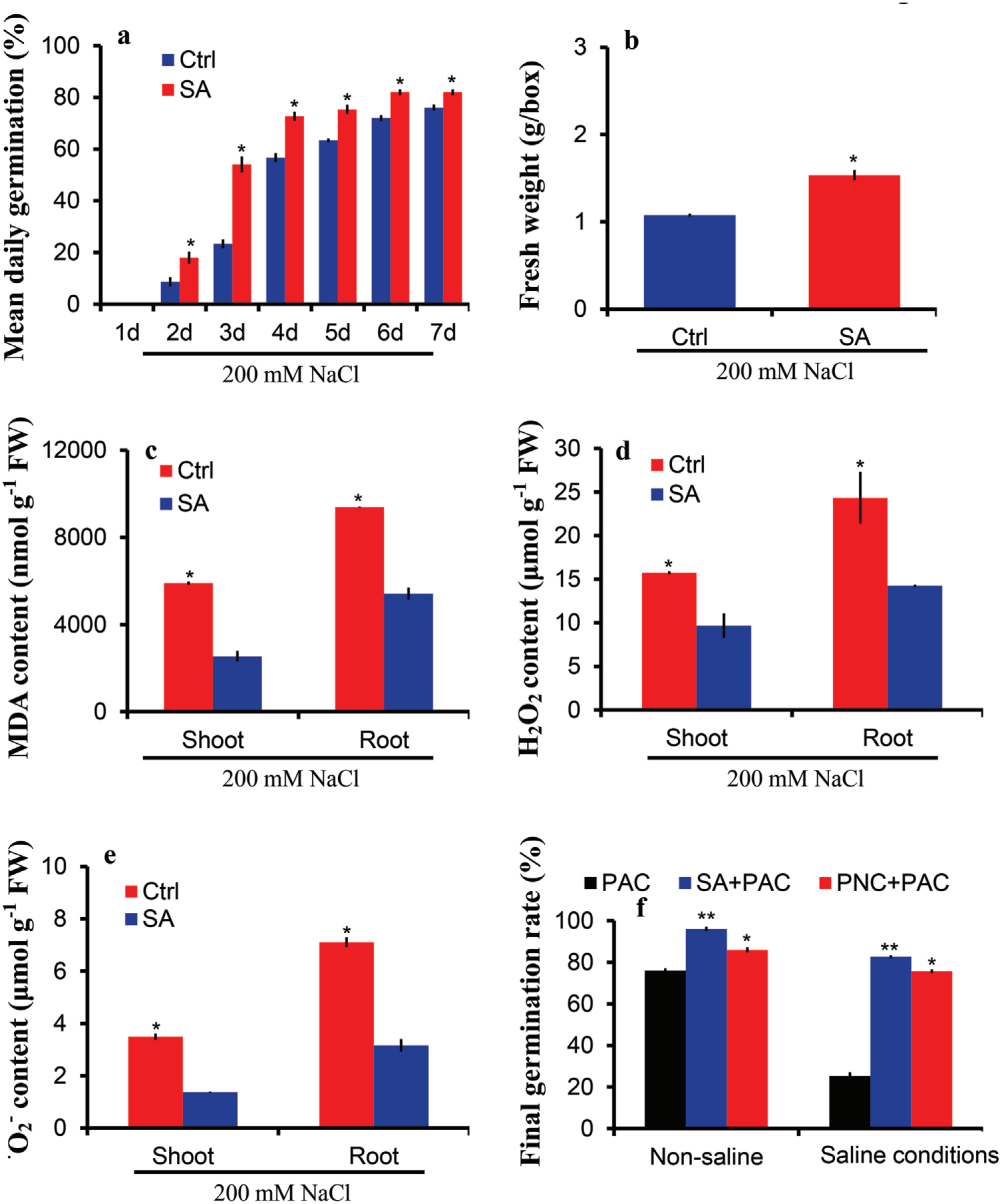
Exogenously supplied SA improved seed germination and biomass of rapeseed under salinity. a) SA seed priming improved germination rate of rapeseed under saline growth conditions, b) SA seed priming increased fresh weight of rapeseed under saline growth conditions, c) seed priming with SA significantly reduced shoot and root MDA content, d) seed priming with SA significantly reduced shoot and root H_2_O_2_ content, e) seed priming with SA significantly reduced shoot and root ^·^O_2_^−^ content in the rapeseed seedlings under saline growth conditions, and f) effect of PAC priming alone or combined with SA and PNC on rapeseed germination under saline or non‐saline growth conditions (7 DAS). The significance between the different treatments at *P* < 0.05 is indicated with * on the vertical bars. One batch was taken as one biological replicate (*n* = 3), while the error bars show the standard error of three biological replicates.

SA exhibited significant differences in shoot and root and priming hours (Figure S4, Supporting Information) among the studied hormones in this experiment. Therefore, a separate batch of the investigation was conducted in which SA was exogenously applied as seed priming treatment. The results of this study revealed SA priming improved rapeseed salt tolerance (**Figure** **5**a–e). SA priming increased seed germination by 8% (82 ± 1.5 vs 72 ± 1.1) under salt stress conditions compared to the control group (Figure 5a). Under salt stress, seed priming with SA increased the fresh weight of rapeseed by 42.5% (1.53 ± 0.05 vs 1.07 ± 0.08 g per box FW) compared to the control group (Figure 5b). There was a decrease of 56.9% (2539 ± 252 vs 5902 ± 74 nmol g^–1^ FW) and 42.3% (5414 ± 274 vs 9391 ± 25 nmol g^–1^ FW) in shoot and root MDA, respectively, for SA priming, compared to the control group under 200 × 10^−3^ m NaCl stress (Figure 5c). Nonetheless, under salt stress conditions, a reduction in the shoot (9.6 ± 1.4 vs 15.7 ± 0.15, µmol g^–1^ FW) and root (14.2 ± 0.1 vs 24.3 ± 2.9 µmol g^–1^ FW) H_2_O_2_ content was observed for SA priming as compared to the control group (Figure 5d). Similarly, 60.7% (1.3 ± 0.02 vs 3.4 ± 0.12 µmol g^–1^ FW) and 55.4% (3.1 ± 0.2 vs 7.1 ± 0.1 µmol g^–1^ FW) decrease in shoot and root ^•^O_2_^−^ the content was observed for SA priming and the control group, respectively under salt stress conditions (Figure 5e). However, no significant differences were recorded for germination rate, fresh weight, root and shoot MDA, H_2_O_2,_ and ^•^O_2_^−^ contents between SA and the control group under normal conditions (Figure S5a–e, Supporting Information). Furthermore, PAC, which is an inhibitor of SA, was used to inhibit the effect of SA concerning rapeseed seed germination. The treatment studied in this experiment were PAC, SA+PAC, and PNC+PAC under non‐saline and saline conditions. Interestingly, PAC inhibited the germination of rapeseed even under normal conditions. Only 76% of seeds were germinated in PAC treatment as compared to SA+PAC (96%) and PNC+PAC (86%) treatments (Figure 5f). However, a drastic inhibition of rapeseed seed germination was recorded for PAC treatment in which 25% of seeds were germinated under saline conditions. Interestingly, SA+PAC and PNC+PAC increased rapeseed germinations by 226% and 198%, respectively, compared to PAC treatment under saline conditions (Figure 5f).

### 2.6 PNC Priming Upregulated the Relative Expression Level of Salicylic Acid Biosynthesis‐Related Genes in Shoot and Root to Cope with Salt Stress

**Figure 6:**
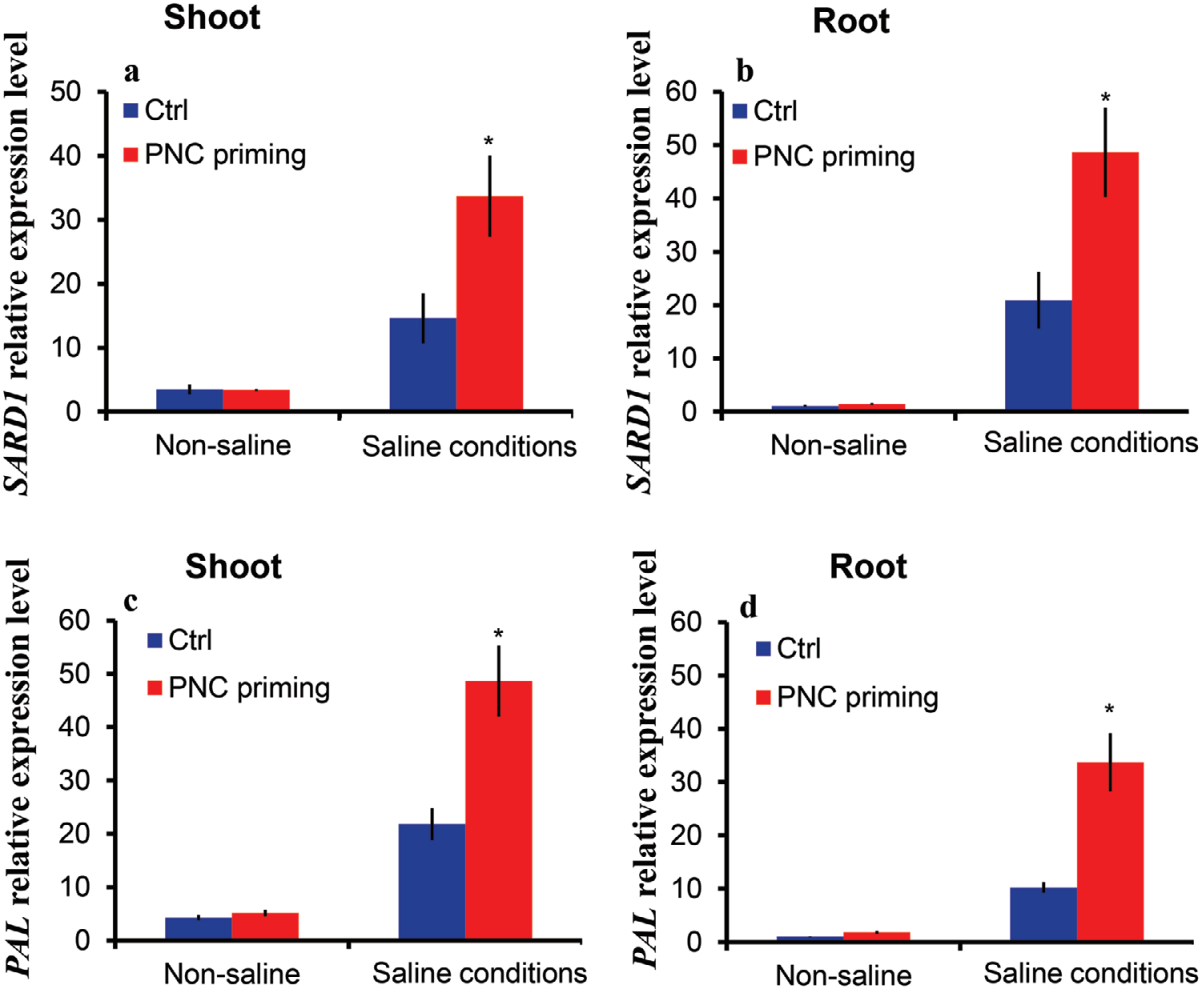
PNC nanopriming up‐regulated the relative expression level of *SARD1* and *PAL* genes. a,b) PNC nanopriming up‐regulated the relative expression level of *SARD1* genes in shoot and root of rapeseed seedlings established from PNC nanopriming under salt growth conditions, c,d) PNC significantly increased the relative expression level of *PAL* genes in shoot and root of rapeseed seedlings established from PNC nanopriming under salt growth conditions. * represents a significant difference between the treatments based on a two‐tailed *t*‐test at 0.05 level. One batch was taken as one biological replicate (*n* = 3), while the error bars show the standard error of three biological replicates.

*SARD1* and *PAL* genes are related to salicylic acid (SA) biosynthesis in plants. Therefore, in our experiment, we determined the relative expression level of the *SARD1* and *PAL* genes for the biosynthesis of SA in the shoot and root of rapeseed seedlings grown under non‐saline and saline conditions. The results from qPCR experiments showed that NNP and PNC priming had no significant effect on the relative expression level of *SARD1* gene in shoot and root of rapeseed seedlings grown under non‐saline conditions (**Figure** **6**a,b). However, under saline conditions, PNC priming upregulated *SARD1* by 130.6% (33.6 ± 6.3 vs 14.6 ± 3.9 relative expression level) and 132.4% (48.6 ± 8.3 vs 20.9 ± 5.3 relative expression level) in shoot and root, respectively, compared to NNP priming. Similarly, no significant difference in shoot and root *PAL* relative expression was observed between NNP and PNC priming under non‐saline conditions (Figure 6c,d). Nevertheless, significantly higher *PAL* gene relative expression level in the shoot (48.6 ± 6.6 vs 21.8 ± 2.9, 122% increase) and root (33.7 ± 5.4 vs 10.2 ± 0.96, 228% increase) of seedlings established from PNC priming compared to the NNP priming was recorded under saline conditions.

## 3 Discussion

### 3.1 Maintaining ROS Homeostasis Could Be a Commonly Employed Mechanism in Nanopriming Improved Plant Salt Tolerance

Nano‐enabled agriculture has emerged as a highly potential approach to increase crop productivity.^[^ ^41^ ^]^ In modern agriculture practices, rapid and uniform seed germination is essential to maintain better seedling establishment, which determines the final yield.^[^ ^7^, ^42^ ^]^ In recent years, nanobiotechnology has been widely adopted in agriculture, including nano‐pesticides,^[^ ^43^ ^]^ nano‐fertilizers,^[^ ^44^ ^]^ nano‐enabled stress tolerance,^[^ ^19^, ^45^ ^]^ and seed nanopriming.^[^ ^19^, ^46^ ^]^ To date, the reported mechanisms regarding nanopriming improved salt tolerance are: 1) decreasing lipid peroxidation,^[^ ^23^ ^]^ 2) increasing a‐amylase activity,^[^ ^46^ ^]^ 3) reducing over‐accumulation of ROS,^[^ ^40^, ^46^, ^47^ ^]^ 4) increasing the activities of antioxidants such as SOD, POD, CAT, and APX,^[^ ^46^, ^47^, ^48^ ^]^ 5) affecting phytohormones balance by stimulating indole‐3‐butyric acid, 1‐naphthalene acetic acid, 6‐benzylaminopurine contents and reducing abscisic acid content,^[^ ^28^ ^]^ 6) increasing chlorophyll content,^[^ ^49^ ^]^ 7) modulating the expression levels of salt stress genes such as upregulation of *P5CS* (delta‐1‐pyrroline‐5‐carboxylate synthetase, key enzyme for proline biosynthesis) and downregulation of *RBOH1* (respiratory burst oxidase, responsible for producing H_2_O_2_ at apoplast),^[^ ^50^ ^]^ and 8) maintaining Na^+^/K^+^ ratio.^[^ ^46^, ^47^ ^]^ Among these reported mechanisms underlying nanopriming improved plant salt tolerance, the majority are associated with the scavenging of over‐accumulated ROS.

Keep a balance between ROS generation and scavenging is important for plant stress tolerance. ROS generation under environmental stress conditions exceeds the scavenging capacity of cellular redox potential, resulting in oxidative stress.^[^ ^39^, ^51^, ^52^, ^53^ ^]^ ROS over‐production poses a significant threat to cells via enzymes inhibition, lipids peroxidation, proteins oxidation, damaging nucleic acids, and initiation of programmed cell death (PCD pathway), and consequently leads to cell death.^[^ ^54^, ^55^, ^56^ ^]^ Thus, plant ability to maintain ROS homeostasis is associated with its stress tolerance.^[^ ^57^, ^58^, ^59^ ^]^ Indeed, nanopriming improved plant salt tolerance is always associated with the increased ROS scavenging ability. For example, cotton seeds soaked with PNC showed significantly reduced ROS content than the control under salinity stress.^[^ ^40^ ^]^ Under salt stress, Ag NPs decreased oxidative stress by improving antioxidant enzyme activity in pearl millet leaves.^[^ ^60^ ^]^ Similarly, Si NPs priming improved sweet pea salt tolerance by enabling better ROS scavenging abilities.^[^ ^61^ ^]^ Here, we found that salt stress increased the accumulation of ROS such as H_2_O_2_ and ^•^O_2_^−^ in rapeseed (Figure 2a,b,d,e). This is in accordance with previous studies showing over‐accumulation of ROS in salt stressed plants.^[^ ^62^, ^63^, ^64^ ^]^ While, PNC priming significantly reduced the intensities of DCF (indicating H_2_O_2_, 94.3% for leaf and 38.4% for root) and DHE (indicating ^•^O_2_^−^, 56.4% for leaf and 41.3% for root) in leaf by 94.3% and 56.4%, respectively, as compared to NNP (non‐nanoparticle control) group under saline conditions (Figure 2c,f). These results confirmed that PNC nanopriming improved rapeseed salt tolerance is associated with the increased ROS scavenging ability. Overall, combined with previous studies, our results suggest that maintaining ROS homeostasis could be a commonly employed mechanism in nanopriming improved plant salt tolerance.

### 3.2 PNC Nanopriming Improved Rapeseed Salt Tolerance Is Associated with the Modulation of Phytohormones

Improvement in salt tolerance with the application of nanoceria has been reported in rapeseed,^[^ ^37^ ^]^ *Moldavian Balm*,^[^ ^65^ ^]^ cotton,^[^ ^66^ ^]^ and *Arabidopsis*.^[^ ^39^ ^]^ Overall, the former studies proposed different nano‐induced salt‐tolerance mechanisms, i.e., improving α‐amylase activities,^[^ ^21^, ^46^ ^]^ better scavenging of ROS,^[^ ^40^, ^67^, ^68^ ^]^ translocation of more Na^+^ to shoot by shortening the root apoplastic barriers,^[^ ^69^ ^]^ enhancing shoot Na^+^ exclusion,^[^ ^66^ ^]^ better maintenance of ROS homeostasis and Na^+^/K^+^ ratio,^[^ ^46^ ^]^ and enabling better mesophyll K^+^ retention.^[^ ^39^ ^]^ Phytohormones are organic materials produced during plant metabolism, capable of regulating physiological responses to mediate plant growth and development.^[^ ^70^ ^]^ Phytohormones determine the abilities of plant adaptation to abiotic stresses by modulating a range of stress‐adaptive responses^[^ ^71^, ^72^, ^73^, ^74^ ^]^ To date, there is limited information provided on how nanopriming modulates plant endogenous hormones to combat hostile environmental conditions. Herein, our results showed that PNC priming significantly increased shoot GA_3_ and ABA contents by 31% and 149%, respectively, compared to NNP treatment under saline conditions (Figure 3a,b). This is in accordance with previous studies that showed Ag NPs nanopriming improved GA and ABA content. While, in wheat, the ABA content was reduced in Ag NPs primed plants than in the water primed control under salt stress.^[^ ^28^, ^75^ ^]^ These controversial results might be associated with a variation in plant species and nanoparticles. Under salinity, compared with no significant difference in shoot IAA and JA content, the PNC nanopriming group showed significantly higher SA content than the NNP priming group (Figure 3c–e). In the root, compared with no significant difference in JA content, PNC nanopriming group showed significantly higher IAA and SA content than the NNP priming group under salinity stress (Figure 4c–e). These results suggest the importance of SA in PNC nanopriming improved rapeseed salt tolerance. Previous studies showed that hydroponically root applied CeO_2_ NPs (characterization data not reported) had no significant effect on the leaves ABA and IAA content in Bt‐transgenic and conventional cotton compared to the control group.^[^ ^30^ ^]^ Here, under non saline condition, PNC nanopriming group showed a significant increase of shoot ABA and JA. The possible reasons could be associated with different application methods, varied properties of nanoparticles, and their dosage, growth stages, and plant species. This further illustrates the complexity of the role of phytohormones in PNC nanopriming improved rapeseed salt tolerance. Together, our results suggest that modulating of phytohormones, i.e., GA, ABA, and SA is important to PNC nanopriming improved rapeseed salt tolerance.

### 3.3 Salicylic Acid Plays an Important Role in PNC Nanopriming Improved Rapeseed Salt Tolerance

Salicylic acid plays an important role in plant salt stress response. Exogenous application of SA is known to improve plant salt tolerance.^[^ ^76^, ^77^, ^78^ ^]^ SA reduced the salt‐induced oxidative stress in mung bean through decreasing MDA content and enabling ROS scavenging.^[^ ^79^, ^80^ ^]^ SA has been widely reported to modulate antioxidant enzymes such as SOD, POD, CAT, GR, and APX for scavenging ROS under various stress conditions.^[^ ^81^, ^82^, ^83^ ^]^ Seed priming with SA increased seed germination of *Leymus chinensis* under salt stress.^[^ ^33^ ^]^ Similarly, here, we showed that SA priming significantly improved rapeseed salt tolerance by having high germination rate, biomass, and low MDA and ROS levels than the water primed control under salinity (Figure 5a–e). While the possible role of SA in PNC nanopriming improved salt tolerance is still obscure and is worthy to be investigated. In this study, we found that compared with NNP priming group, shoot SA content in PNC nanopriming group was decreased under non‐saline conditions while increased under salinity (Figure 3e). It suggests that SA could play a role in PNC nanopriming improved rapeseed salt tolerance. This is further confirmed by the results that compared with no difference under non‐saline conditions, PNC nanopriming group has significantly higher root SA content than the NNP priming group under salinity (Figure 4e). No doubt, increased SA content is always associated with plant salt tolerance.^[^ ^84^, ^85^ ^]^ Also, salt tolerant varieties have significantly higher SA content than the sensitive ones.^[^ ^86^, ^87^, ^88^ ^]^

To further validate the role of SA in PNC nanopriming improved rapeseed salt tolerance, SA inhibitor PAC was used. Our results showed that compared with PAC treatment, PNC+PAC showed significantly increased germination rate under non‐saline and salinity conditions (Figure 5f). It suggests that PNC priming helped to increase SA content in rapeseeds. This is in accordance with our previous data (Figures 3e and 4e), showing that under salinity stress, PNC nanopriming group had significantly higher SA content in shoot and root than the NNP priming control. Also, under salinity stress, the expression level of SA biosynthesis related genes *SARD1* and *PAL* are upregulated in PNC than NNP nanopriming group (Figure 6a–d). A previous study stated that SAR DEFICIENT1 (*SARD1*) regulates the expression level of *ICS1*, which is essential for the pathogen induction of SA biosynthesis.^[^ ^89^ ^]^ Several other studies further supported these findings documenting that in *SARD1* mutants, the synthesis of SA was blocked,^[^ ^90^, ^91^ ^]^ while the plants were highly susceptible to stress.^[^ ^89^, ^91^ ^]^ Similarly, *PAL* genes have long been reported to contribute to the biosynthesis of SA under stress conditions in different crops species such as tobacco,^[^ ^92^ ^]^ rice,^[^ ^93^ ^]^ *Arabidopsis*,^[^ ^94^ ^]^ and soybean.^[^ ^95^ ^]^ Moreover, it is evident from the previous studies that the regulation of SA biosynthesis by *SARD1* and *PAL* pathways plays a vital role in plant stress tolerance. Under salinity stress, upregulation of SA biosynthesis related genes is always associated with plant salt tolerance.^[^ ^96^, ^97^ ^]^ Also, overexpression of SA biosynthesis related genes increased SA content and improved plant salt tolerance.^[^ ^98^, ^99^ ^]^ SA‐deficiency in plants was considered a major reason for salinity‐induced damages and reduced activity of antioxidant enzymes in SA‐deficient *NahG* transgenic *Arabidopsis* lines.^[^ ^100^ ^]^ Interestingly, under non‐saline condition, decreased shoot SA content (Figure 3e) were found in PNC than NNP nanopriming group, and no difference in root SA content (Figure 4e) and expression level of *SARD1* and *PAL* in shoot and root (Figure 6a–d) was found between PNC and NNP nanopriming groups. Combined with the results shown in Figure 5f, it suggests that besides SA, other factors are involved in PNC nanopriming improved rapeseed salt tolerance. Indeed, our previous data (Figure 2) showed that PNC enabled ROS scavenging is an important mechanism underlying PNC nanopriming improved rapeseed salt tolerance. It suggests that the coordination between SA level and ROS scavenging could be important for PNC nanopriming improved rapeseed salt tolerance. Overall, our results suggest that under salinity stress, besides PNC increased ROS scavenging ability, SA plays an important role in PNC nanopriming improved rapeseed salt tolerance. Also, the coordination between different employed mechanisms could be of importance for nanopriming improved plant stress tolerance.

## 4 Conclusion

With the advantages of low usage (less concern about biosafety) and low cost, seed priming with nanoceria becomes a promising approach in improving plant salt stress tolerance. However, less known is the mechanisms underlying nanoceria nanopriming improved plant salt tolerance. In this study, we found that besides the increased ROS scavenging ability, nanoceria priming improved rapeseed salt tolerance is associated with the modulation of salicylic acid level. Our results add more knowledge about the mechanisms behind nanoceria priming improved plant salt tolerance. Future studies are encouraged to investigate the role of salicylic acid and its coordination with other mechanisms in nanopriming improved plant stress tolerance.

## 5 Experimental Section

### The Synthesis and Characterization of Nanoceria (PNC)

In a 50 mL conical tube, 4.5 g poly (acrylic) acid (1800 MW, Sigma Aldrich, Lot No. SLCG7887) and 1.08 g cerium (III) nitrate (Sigma Aldrich, 99 percent, Lot No. BCCB4305) were dissolved in 5 mL and 2.5 mL ddH_2_O, respectively, as described in our previous study.^[^ ^40^ ^]^ These solutions were vigorously mixed for 15 minutes in a vortex mixer at 2000 rpm. In a 50 mL small beaker, 15 mL of 30% ammonium hydroxide solution (Sigma Aldrich, 7.2 M, Lot No. STBJ8789) was added. Dropwise additions of poly (acrylic) acid and cerium (III) nitrate were made to the ammonium hydroxide solution with constant stirring at 500 rpm in a fume hood overnight at room temperature. After 24 h, the debris and oversized agglomerates were removed, and the resultant solution was transferred to a 50 mL conical tube and centrifuged for one hour at 4000 x g. For further purification, the resulting supernatant was transferred to three 15 mL 10 kDa filters (MWCO 10 K, Millipore Inc., Lot No. RIEB08895) and centrifuged at 4500 rpm for six cycles (45 min each cycle). While recording the absorbance at 271 nm with a UV‐VIS spectrophotometer (UV 1800PC, AOE, Shanghai, China), Beer Lambert's law was used to calculate the concentration of the synthesized PNC solution (Figure S1, Supporting Information). A dynamic light scattering apparatus (Malvern Zetasizer, Nano) was used to record the size, and zeta potential of PNC dispersed in DI water. For transmission electron microscopy (TEM) imaging, 0.45 × 10^−3^ m PNC nanoparticles were dispersed in ethanol. The TEM samples were put on pure C grids with a Cu mesh of 200 mesh (0 1840, Ted Pella Inc.). An FEI Talos microscope running at 300 kV was used to obtain TEM images of three individual samples.

### Seed Materials, Seed Priming, Stress Treatments, and Growth Conditions

In this experiment, salt‐sensitive rapeseed variety “Zhongshuang 11” (ZS 11) was used as seed material.^[^ ^10^ ^]^ Based on our previous study, 0.1 × 10^−3^ m was the optimum concentration of PNC.^[^ ^46^ ^]^ Therefore, 0.1 × 10^−3^ m PNC was used to prime seeds in this study. 10 × 10^−3^ m of TES buffer (pH 7.5, regulated by HCl) was used to dissolve PNC.^[^ ^45^ ^]^ As a no‐nanoparticle control (NNP), 10 × 10^−3^ m TES buffer was used. PNC+TES or TES solutions were used to prime the seeds. Conical flasks were added with a seed to solution ratio of 1:5 (w/v)^[^ ^7^ ^]^ and were put on a mechanical shaker (SLK‐03000‐S, SCILOGEX, USA) (50 rpm) for 8 h in dark conditions. After 8 h of priming, the seeds were surface washed with DI water and were kept to dry back in the dark at room temperature. Then the seeds were sown in 12×12×6 cm polyethylene boxes in length, width, and height. The germination boxes were placed with three sterilized germination papers, and 10 mL of DI water or 200 × 10^−3^ m NaCl solution was added. Every other day, the bottom two germination papers were replaced with two new papers, and the boxes were added with 7 mL of DI water or salt solution. The growth chamber environment was set as 14 h of light (200 mol m^−2^ s^−1^) and 10 h dark with 25 ± 1 and 20 ± 1 °C temperatures. 7 days after sowing (DAS), rapeseed seedlings were divided into shoot and root, and the lengths were measured with a ruler. The dry biomass was recorded by oven‐drying at 75 °C.

### Monitoring ROS Scavenging by PNC

The dyes 25 × 10^−6^ m 2′,7′‐dichlorodihydrofluorescein diacetate (H2DCFDA, Thermo Fisher Scientific, Lot No. D399) (in TES infiltration buffer, pH 7.5) and 10 × 10^−6^ m dihydroethidium (DHE, Thermo Fisher Scientific, Lot No. D23107) (in TES infiltration buffer, pH 7.5) were used to incubate cotyledon discs and roots of rapeseed seedlings separately in 1.5 mL Eppendorf tubes. The tubes were covered with aluminum foil to protect the samples from light. DCF (2′,7′‐ dichlorofluorescein) and DHE (fluorescent product 2‐hydroxyethidium) were used to measure the in vivo contents of H_2_O_2_ and ^•^O_2_^−^, respectively. The samples were mounted on the glass slides after staining with DCF and DHE dyes. For better confocal imaging, a drop of perfluorodecalin (PFD, Lot No. 2 021 121) was poured into each slice. A square coverslip was carefully pushed onto the mounted sample to cover the sample completely. Leica laser scanning confocal microscope (TCS, SP8) was used to image the prepared sample slides. The confocal microscope in the cotyledon discs was focused on chloroplasts, whereas the confocal microscope in the roots was focused on root cells. The following are the imaging settings: Z‐Stack section thickness: 4 m; 488 nm laser excitation; PMT1, 500–600 nm; PMT2, 700–800 nm. Confocal microscopy was performed on 3–4 biological replicates. LAS AF Lite was used to measure the fluorescence intensity of DHE and DCF.

### Determination of Hormones Concentration in Rapeseed Seeds and Seedlings

According to the previous protocols of Liu et al.,^[^ ^101^ ^]^ briefly, 0.05 g seed sample (during the priming hours) or root and shoot sample (post‐germination) were ground with liquid nitrogen. The ground powder was transferred to the Eppendorf tube, and 750 µL extract liquid buffer was added (including internal standard, components: methanol: ddH_2_O: Acetic acid = 80:19:1). The samples were kept for shaking (150 rpm min^−1^, 4 °C) overnight, followed by centrifugation at 13000 rpm for 15 min at 4 °C. The samples were transferred into a brown bottle (200 µL) with the help of a 1 mL syringe containing 0.22 × 10^−6^ m filter membrane (Jin Teng company nylon 66, Lot No. RIEB95060). The standard used for the determination of different hormones concentration were as follow: ^2^H_5_‐IAA as IAA internal standard, 10 ng mL^−1^, Olchemlm Ltd CAS: 76937‐78‐5, ^2^H_6_‐ABA as an ABA internal standard, 10 ng mL^−1^, Olchemlm Ltd CAS: 35671‐08‐0, (±) ‐9, 10‐DIHYDROJASMONIC ACID as JA, 10 ng mL^−1^, Olchemlm Ltd, CAS: 3572‐64‐3, and N‐acetyl aspartic acid (NAA) as the internal standard 100 ng mL^−1^ of SA, sigma CAS number 86‐87‐3. The standard was diluted with methanol (methanol concentration is consistent with the sample constant volume) with the order: 0.25 ppb, 0.5 ppb, 1 ppb, 2 ppb, 5 ppb, 10 ppb, and 20 ppb.

Gibberellic acid (GA_3_) concentration was determined using the ELISA kit provided by the MEIBIAO BIOLOGY company (Item number: 202 105). About 0.05 g seed or root and shoot samples were ground with phosphate buffer (pH 7.5) and centrifuged at 13000 at 4 °C to obtain fine supernatant. The GA_3_ concentration was quantified by following the standard procedure provided by the manufacturer. Finally, the reading was recorded at 450 nm using a microliter plate reader.

### Quantification of MDA and ROS Contents

The malondialdehyde (MDA) content was measured following standard methods detailed in a previous study.^[^ ^7^ ^]^ A UV–vis spectrophotometer was used to measure the absorbance at 450, 532, and 600 nm (UV 1800PC, AOE, Shanghai, China). The MDA concentration (mol L^−1^) was estimated as: MDA concentration (mol L^−1^) = 6.45 (A532‐A600)‐0.56A450, where A stands for absorbance at various wavelengths. Finally, MDA content (mol g^−1^) = C V/(1000 W), where C is the MDA concentration, V is the sample extraction liquid (mL), and W is the sample weight. Quantification of superoxide anion (^•^O_2_^−^) and hydrogen peroxide (H_2_O_2_) and was done using kits from “Solarbio Life Sciences (Item number: 20 210 903),” and “Nanjing Jiancheng Biotechnology Co., Ltd (item number: A04‐1‐1)” respectively. The contents of H_2_O_2_ and ^•^O_2_^−^ were estimated using the manuals given by the individual manufacturers.

### Seed Priming with Exogenously Applied Salicylic Acid

Seedlings established from PNC priming showed significant differences in shoot and root SA concentrations among the studied hormones under non‐saline and saline conditions. Furthermore, PNC priming modulated the concentration of endogenous hormones during the 3h and 8h priming hours. Therefore, SA was exogenously applied as a priming treatment to validate the effect of SA on rapeseed salt tolerance. The concentration of exogenously supplied SA (150 mg L^−1^) was based on SA concentration during 8h PNC priming.

Firstly, the seeds were hydro‐priming or TES primed for three hours. As the concentration of hormones was significantly influenced by PNC priming at 3h, the TES solution was replaced with SA+TES solution, and hydro‐priming treatment was kept as a control check. The germination rate was monitored daily. Seedlings were picked and surface washed with ddH_2_O, followed by blotted drying with paper after 7 days of sowing seeds in salt or normal growth conditions. Following the measurement of fresh seedling weight, the seedlings were dissected into shoots and roots. About 0.1 g shoot and root samples were weighted and immediately transferred into a liquid nitrogen tank before moving to a −80 °C refrigerator until further use.

### Inhibition of SA Synthesis via Paclobutrazol

To further validate the PNC‐regulated biosynthesis of SA, paclobutrazol (PAC, a SA inhibitor, item no. 46046‐250MG, Sigma, 15 mg L^−1^) was used to inhibit the biosynthesis of SA. In this experiment, the treatments tested were seed priming with PAC, PNC+PAC, and PAC+SA. The primed seeds were sown under non‐saline and 200 × 10^−3^ m NaCl stress.

### Isolation of RNA and Quantitative Real‐Time PCR (qRT‐PCR) Analysis

Total RNA was isolated using the RNAprep pure Plant Kit (DP432, Tiangen, Beijing, China, Lot No. RN53). 2 g of total RNA was reverse transcribed into cDNA using the TRUEscript First Strand cDNA Synthesis Kit (PC5801, Aidlab, Beijing, China). The amplification of qRT‐PCR products was performed according to the manufacturer's instructions in a reaction mixture of 10L 2SYbr Green qPCR Mix (PC3301, Aidlab, Beijing, China). The Bio‐Rad CFX Connect Real‐Time PCR System was used for the qRT‐PCR analysis (Bio‐Rad, California, USA). Three biological and three technical replicates were used for each treatment. The 2^−ΔΔCt^ technique was used to calculate relative gene expression. The primers used for qRT‐PCR are supplemented in Table S1 in the Supporting Information.

### Statistical Analysis

The Independent‐Samples T‐Test in SPSS software (23.0) was used to compare means. Non‐parametric tests based on 1‐Sample K‐S (Kolmogorov‐Smirnov test) were used to test the normal distribution of all data. * denotes significance level at *p* < 0.05. Standard errors are shown by error bars (*n* = 3). Excel 2016 was used to create the graphs.

## Conflict of Interest

The authors declare no conflict of interest.

## Supporting information

Supporting Information

## Data Availability

The data that support the findings of this study are available from the corresponding author upon reasonable request.
