# Supplementary material for: CeO2 Nanoparticles Seed Priming Increases Salicylic Acid Level and ROS Scavenging Ability to Improve Rapeseed Salt Tolerance
Source: Glob Chall. 2022 May 19;6(7):2200025. doi: 10.1002/gch2.202200025 (PMC9284644; doi:10.1002/gch2.202200025)
Supplement: Supplementary file 1 — Supporting Information [file GCH2-6-2200025-s001.pdf]

Global Challenges
Open Access

Supporting Information

for *Global Challenges,* DOI: 10.1002/gch2.202200025

CeO_2 Nanoparticles Seed Priming Increases Salicylic Acid Level and ROS Scavenging Ability to Improve Rapeseed Salt Tolerance

Mohammad Nauman Khan, Yanhui Li, Chengcheng Fu, Jin Hu, Linlin Chen, Jiasen Yan, Zaid Khan, Honghong Wu, and Zhaohu Li**

CeO_2 nanoparticles seed priming increases salicylic acid level and ROS scavenging ability to improve rapeseed salt tolerance

Mohammad Nauman Khan[1], Yanhui Li[1], Chengcheng Fu[1], Jin Hu[1], Linlin Chen[1], Jiasen Yan[1], Zaid Khan[1], Honghong Wu[1, 2, 3,]*, Zhaohu Li[1, 2,3,]*

[1] MOA Key Laboratory of Crop Ecophysiology and Farming System in the Middle Reaches of the Yangtze River, College of Plant Science and Technology, Huazhong Agricultural University, Wuhan, China 430070

[2] Hongshan Laboratory, Wuhan, Hubei, China 430070

[3] College of Agronomy and Biotechnology, China Agricultural University, Beijing, China 100083

* Correspondence: honghong.wu@mail.hzau.edu.cn; lizhaohu@cau.edu.cn

Supporting information

Table S1: Primers used in this study.

**Genes names**	**Sense primers (5′-3′)**	**Antisense primers (5′-3′)**
SARD1	CGGTTTGTGAAGCGATGACC	CTTGGCCCAAAATTTGACGGA
PAL	TGGATGAAGTGAAGAGAATGGT	ACACCATAACTATCAGTGCCT
Actin	CTGACCGTATGAGCAAAG	CCACCGAACCAGAAGGCAGA

Figure S1

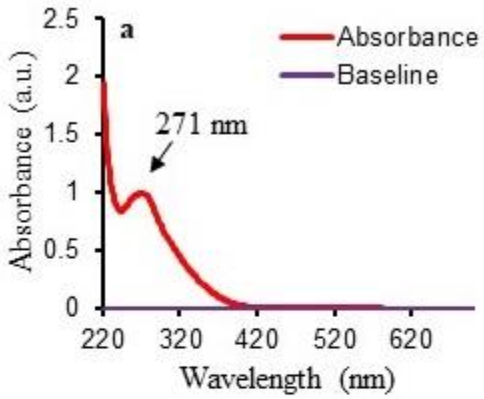

Figure S1. Absorbance of PNC showing a clear peak at 271 nm.

Figure S2

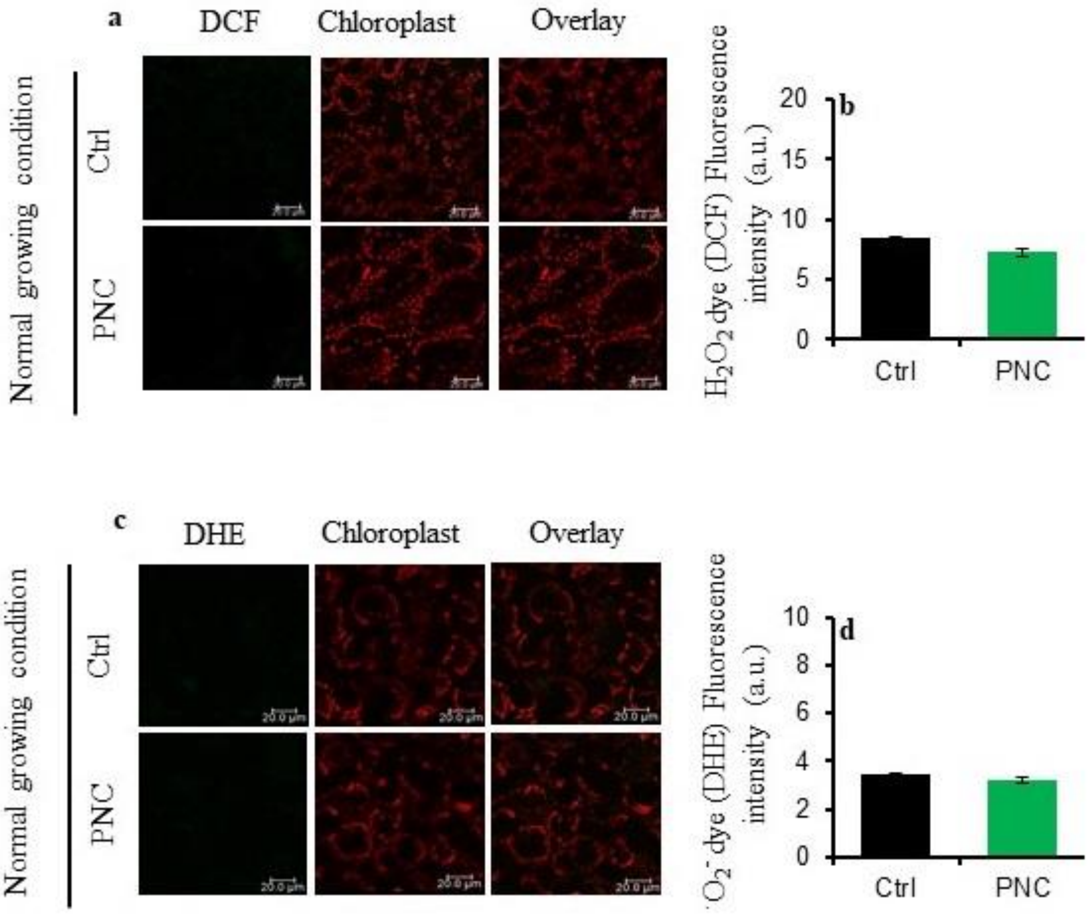

Figure S2. ROS level in leaf cells of PNC primed rapeseed under non-saline condition. (a) confocal imaging of hydrogen peroxide (H_2O_2) scavenging in leaf cells of rapeseed seedling leaves by PNC under non-saline growing conditions, **(b)** the fluorescence intensity of H_2O_2 dye (DCF) in leaf cells of rapeseed seedling leaves under non-saline growing conditions **(c)**, confocal imaging of superoxide anion ($^{\bullet}O_2^{-}$) scavenging in leaf cells of rapeseed seedling leaves by PNC under non-saline growing conditions, and **(d)** the fluorescence intensity of $^{\bullet}O_2^{-}$ dye (DHE) in leaf cells of rapeseed seedling leaves under non-saline growing conditions. The significance between the different treatments at $P < 0.05$ is indicated with * on the vertical bars. Scale bar: 20 µm.

Figure S3

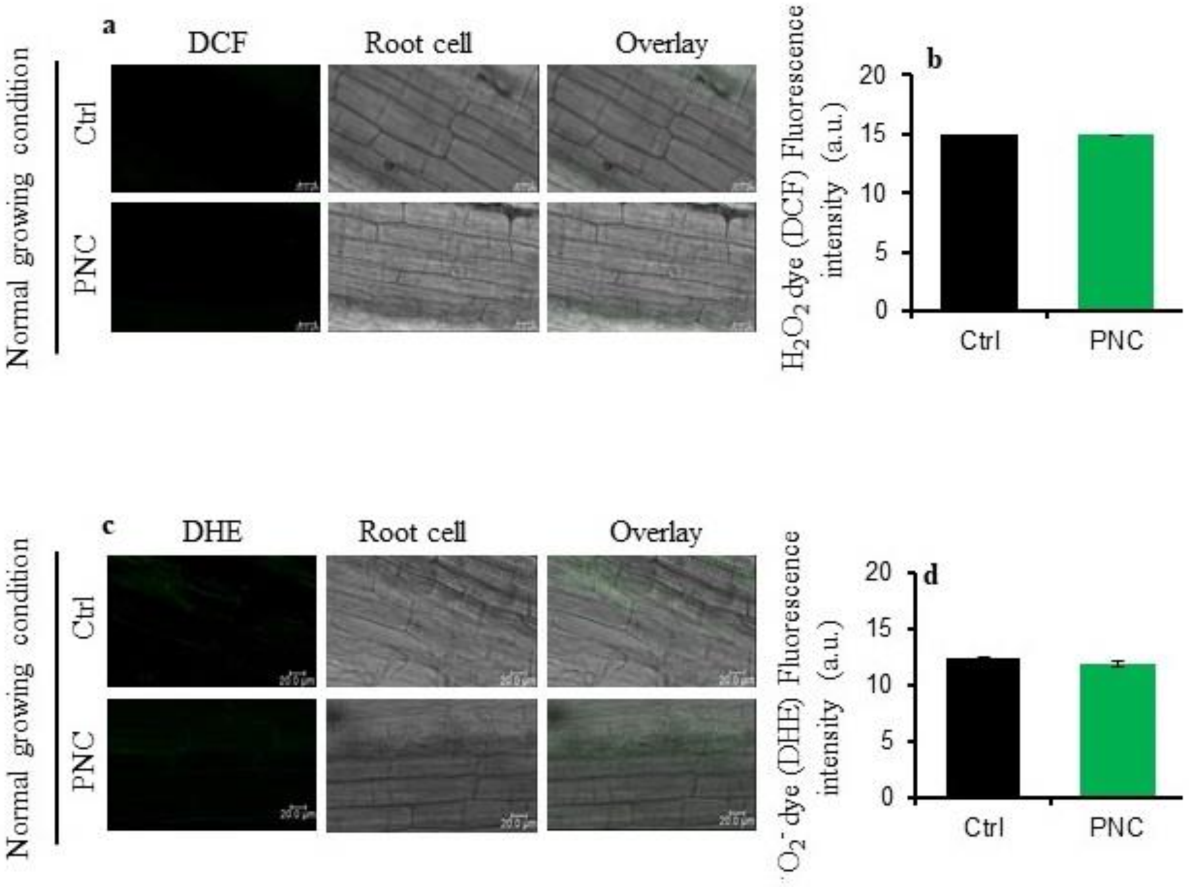

Figure S3. ROS level in root cells of PNC primed rapeseed under non-saline condition. (a) confocal imaging of hydrogen peroxide (H_2O_2) scavenging in root cells of rapeseed seedlings by PNC under non-saline growing conditions, **(b)** the fluorescence intensity of H_2O_2 dye (DCF) in root cells of rapeseed seedlings under non-saline growing conditions, **(c)** confocal imaging of superoxide anion ($^{\bullet}O_2^{-}$) scavenging in root cells of rapeseed seedlings by PNC under non-saline growing conditions, and **(d)** the fluorescence intensity of $^{\bullet}O_2^{-}$ dye (DHE) in root cells of rapeseed seedlings under non-saline growing conditions. The significance between the different treatments at $P < 0.05$ is indicated with * on the vertical bars. Scale bar: 20 µm.

Figure S4

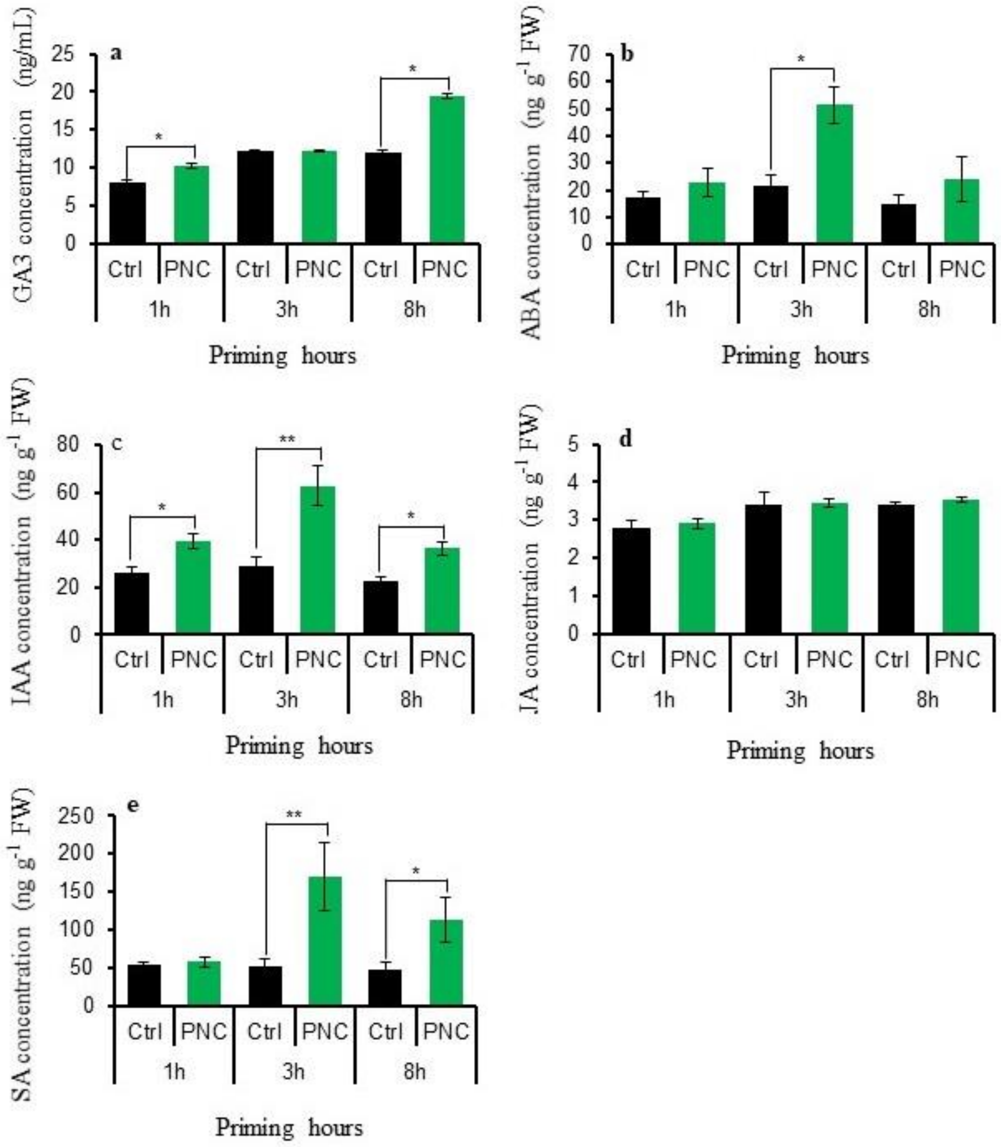

Figure S4. PNC nanopriming modulated seed hormones contents during the imbibition period. (a) GA_3 concentration in seeds primed with or without PNC at 1, 3, and 8 priming hours, **(b)** ABA concentration in seeds primed with or without PNC at 1, 3, and 8 priming hours, **(c)** IAA concentration in seeds primed with or without PNC at 1, 3, and 8 priming hours, **(d)** JA concentration in seeds primed with or without PNC at 1, 3 and 8 priming hours, **(e)** SA concentration in seeds primed with or without PNC at 1, 3, and 8 priming hours. The significance between the different treatments at $P < 0.05$ is indicated with * on the vertical bars. One batch was taken as one biological replicate (n = 3), while the error bars show the standard error of three biological replicates.

Figure S5

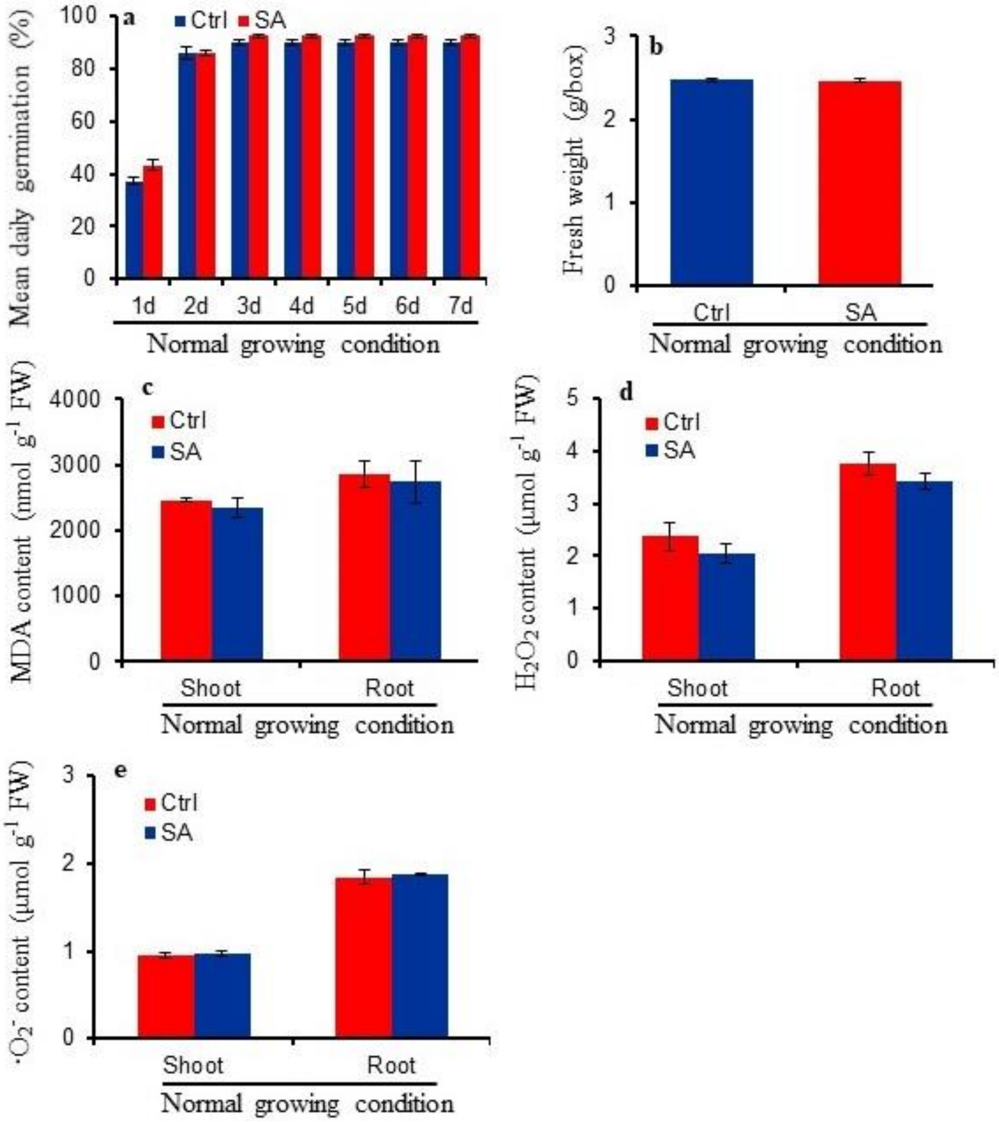

Figure S5. The effect of SA seed priming in rapeseed under non-saline conditions. (a) germination rate of rapeseed under non-saline (7 days) conditions, **(b)** fresh weight of rapeseed under non-saline (7 days) conditions, and **(c, d, e)** MDA, H_2O_2, and $^{\cdot}O_2^{-}$ contents in the rapeseed seedlings shoot and root, respectively, under non-saline (7 days) conditions. The significance between the different treatments at $P < 0.05$ is indicated with * on the vertical bars. One batch was taken as one biological replicate (n = 3), while the error bars show the standard error of three biological replicates.
